# Selective Removal of Malachite Green Dye from Aqueous Solutions by Supported Liquid Membrane Technology

**DOI:** 10.3390/ijerph16183484

**Published:** 2019-09-19

**Authors:** Mohammad Waqar Ashraf, Nidal Abulibdeh, Abdus Salam

**Affiliations:** 1 Department of Mathematics & Natural Sciences, Prince Mohammad Bin Fahd University, Alkhobar 31952, Saudi Arabia; nabulibdah@gmail.com; 2 Department of Chemistry, King Abdulaziz University, Rabigh 21911, Saudi Arabia; slama1971@gmail.com

**Keywords:** liquid membrane, removal, dyes, wastewaters

## Abstract

A lab-scale study on the application of supported liquid membranes (SLM) has been conducted for recovery and selective removal of Malachite Green dye from wastewater. Naturally occurring non-toxic vegetable oils have been used as membrane liquids. Polyvinylidene fluoride (PVDF) films have been used as supports for the liquid membrane. Various parameters affecting the dye permeation such as initial dye concentration, pH, stripping acid concentration, oil viscosity and membrane stability have been investigated. The highest flux value (1.65 × 10^−5^ mg/cm^2^/sec) was obtained with a sunflower oil supported membrane at pH 11 in the feed and 0.25 M HCl in the stripping solution. The membrane showed good stability under optimum conditions and maximum transport was achieved in 8 h of permeation time.

## 1. Introduction

Textile and printing industries discharge huge amounts of effluent which, along with other contaminants, are mostly colored. Elimination of color from wastewaters is a challenging problem. The color comes from the diversity of dyes used in textile and many other industries. By definition, dyes are chemical compounds which are designed to adsorb/absorb or deposit within a surface to impart color. These molecules have the ability to attach themselves onto the surface of cloth to impart color. According to a recent review [1], about 100,000 synthetic dyes exist, with annual production reaching more than 7 × 10^5^ tons per year and they contribute significantly towards water pollution.

Malachite Green (MG) is a cationic dye which is water soluble and used for the dyeing of cotton, wool, paper, leather products, jute, and silk. It is also used as an antiseptic and medical disinfectant. It is resistant to biodegradation and known to cause damage to the brain, kidney and liver when ingested. It is acutely toxic to a wide range of terrestrial and marine animals. Its presence in water bodies reduces photosynthesis by blocking light and drastically affects aquatic ecosystems. In order to remove the dyes from waste effluent, many techniques have been used. These include adsorption, precipitation, membrane filtration, photochemical degradation and electrolytic treatments [2,3,4]. A comprehensive review about the removal of synthetic dyes from wastewater has been published by Forgacs et al. [5]. In addition, Banat et al. [6] have reviewed microbial decolorization of effluent containing dyes. A wide range of treatment technologies have been applied to remove MG from aqueous media, such as photocatalysis [7], biodegradation [8], coagulation [9], solar degradation [10], solvent extraction [11] and sono-photocatalytic degradation [12]. Asfaram et al. have reported a nanoparticle-based micro-solid-phase extraction to assay MG in aqueous samples [13]. Ghaedi et al. have reported ultrasonic-assisted removal of MG dye by ZnO nanorod-loaded activated carbon [14]. Recently, Ruan et al. reported the removal of rhodamine B and crystal violet by using reduced-graphene-oxide-supported nanoparticles [15,16]. They have also reported modeling of MG removal from aqueous solutions by nanoscale zerovalent zinc using artificial neural networks [17].

However, these techniques have shortcomings in terms of effectiveness and cost-efficacy. Supported liquid membrane (SLM) technology has attained commercial and technical success owing to its environmental and eco-friendly industrial applications. Table 1 shows a comparison between some well-known techniques for dye removal [3]. The possibility of using ultra-thin layers of organic solutions, immobilized on micro-porous inert polymeric hydrophobic supports interposed between two aqueous solutions, was first proposed more than two decades ago. Such immobilized liquid layers, representing SLM, for selectively removing chemical species from a mixture have since attracted the interest of several researchers [2,3,4,18]. In principle, the transport of metal species through SLMs can be formally defined as the concurrent combination in a single stage of an extraction and a stripping process, occurring under non-equilibrium conditions. The SLM comprises of a solution of an extracting agent in a water-immiscible low dielectric constant diluent absorbed on a micro-porous polymeric film with a thickness ranging from 25 µm to 50 µm. The polymeric film, which acts as solid support for the liquid membrane, is generally made of polypropylene, polysulfone or other hydrophobic materials and has a pore size ranging from 0.02 µm to 1 µm. The SLM is interposed between two aqueous solutions, formally called feed and strip solutions. A recent literature review indicated the dearth of the use of SLMs for the removal of chemical species from aqueous medium [18]. Also, the reported literature on the use of SLMs requires synthetic solvents and reagents which are mostly toxic and hazardous [1,2,19,20]. Therefore, in the present work, we used non-toxic edible oils as membrane liquids. These oils are glycerides of fatty acids and have other applications like biofuels and biodiesels. A variety of parameters controlling the transport of MG dye have been investigated. These factors include effective pH range in the feed solution, acid concentration in the strip solution, initial dye concentration, selection of vegetable oils and membrane stability.

## 2. Materials and Methods

**Figure 1:**
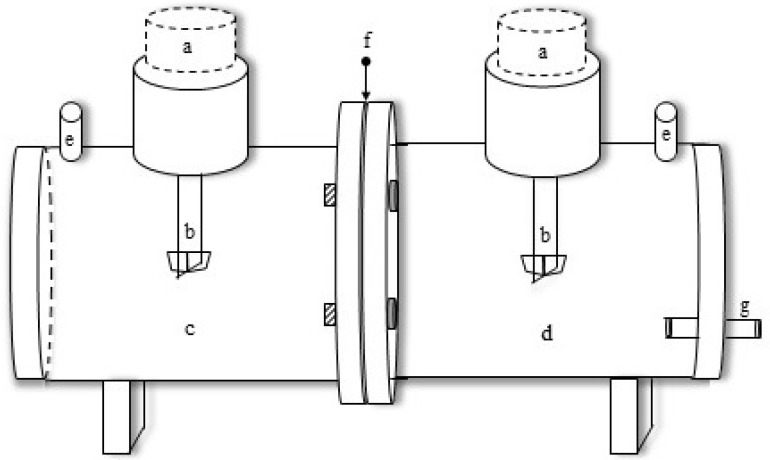
Membrane reactor. **a** Variable speed stirring motors; **b** stirrers; **c** feed chamber; **d** strip chamber; **e** sampling port; **f** oil supported membrane; **g** wastewater outlet.

The vegetable oils used in the present study were procured from local hypermarkets (Afia International Company, Jeddah, Saudi Arabia). Malachite Green dye (formula = C_23_H_25_N_2_; MW = 364.911 g/mol) and hydrochloric acid (AR grade) were purchased from E.Merck (Darmstadt, Germany). Doubly distilled water was used to make all the solutions in the present study. The viscosity of the vegetable oils was determined by using a Brookfield DV1 digital viscometer. The vegetable oils were supported on a micro-porous polyvinylidene fluoride (PVDF) membrane by Durapore (3M, United States). This polymeric support has a thickness of 125 µm, pore size of 0.2 µm, 75% porosity and tortuosity of 1.67. The film is physically tough, chemically resistant and hydrophobic in nature. These characteristics make it an excellent choice to make an SLM. Vegetable oils were impregnated into the membrane by soaking it in the oils for 24 h. Excessive oil was removed with the help of absorbent tissue paper. The prepared membrane was clamped amid the two chambers of the membrane reactor as shown in Figure 1. Dye samples were analyzed on a UV/Vis spectrometer (Shimadzu 5301-PC, Kyoto, Japan) at a wavelength of 616.5 nm. 

The permeation cell (membrane reactor) to carry out separation studies was fabricated with Perspex. This batch-scale permeation cell comprises two chambers of identical volume and shape. The volume of each compartment is 140 mL and a membrane with an effective surface area of 14.2 cm^2^ could be fixed between the two compartments. Each chamber (compartment) is provided with one sample collector opening and another for the variable speed stirrer. Vigorous stirring is required to minimize boundary layer resistance. To run the experiments at specific temperatures, the permeation cell can be placed in a water bath. 

The flux of dye through the SLM is defined as mass transferred per unit area of the SLM, per unit time. The flux (F) was calculated using the following equation [20]:

(1) $$F\;=\;-\frac{dc}{dt}\;\cdot \;\frac{v}{a}$$

where *c* is concentration, *t* is time, *v* is the volume of chamber and *a* is the area of the SLM.

The permeation coefficient (*p*) was calculated using an integrated form of Equation (1):

(2) $$\mathrm{In}\;\frac{c}{c_{o}}\;=\;-\frac{a}{v}\;\cdot \;p.t$$

where *C_o_* is the concentration at time zero. 

All experimental measurements were taken in triplicate and deviations between repetitive experiments were less than 5%.

## 3. Results and Discussion

The different vegetable oils that were selected to be used as membrane liquids for the transportation of MG are presented in Table 2 along with the values for viscosity and flux. These fluxes were obtained with 100 mg/L dye at pH 11 in the feed chamber, 0.25 M HCl in the strip chamber, a stirring speed of 500 rpm and a transportation time of 7 h. The results showed that coconut, sunflower, olive and mustard oils can be used for transport. The highest flux was shown by sunflower oil, followed closely by coconut oil. Olive oil showed lower flux owing to its higher viscosity, which is due to the presence of mixed triglyceride esters of oleic acid, palmitic acid and other fatty acids. Castor oil, by virtue of high viscosity (485 cP), could not be used for transportation. With mustard and groundnut oil, it was observed that dye accumulated at the membrane surface, hindering the transportation process. Therefore, these oils were not considered useful for further permeation studies. 

### 3.1. Role of pH in the Feed Chamber

**Figure 2:**
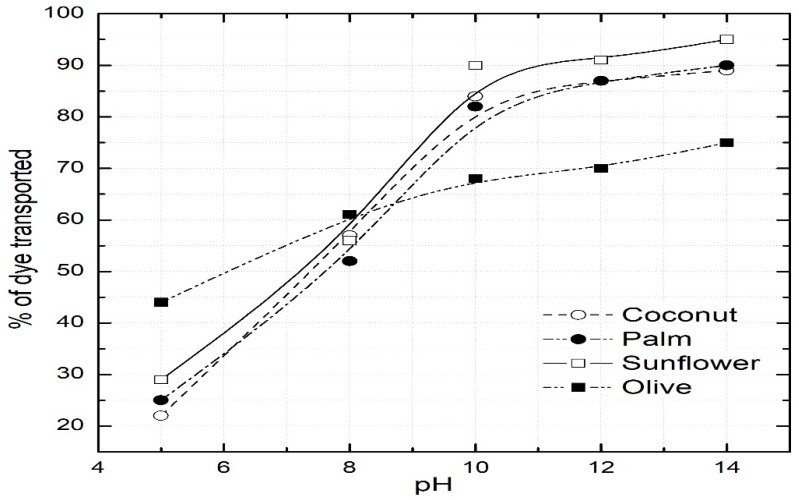
Effect of pH in the feed chamber. Dye concentration = 100 mg/L; strip chamber = 0.25 M HCl; permeation time = 14 h.

The vital role of pH in the feed chamber on the dye transport was studied by performing experiments at a range of pH values. Figure 2 depicts the results along with the experimental conditions. It is observed that the transport of the dye through the SLM increases as the pH value in the feed chamber increases. However, it reaches a maximum at a pH value of 11 ± 0.5. At values higher than 11, the percentage of the dye transported decreases with time. This can be attributed to the fact that at higher pH values, the dye solubility decreases in the oil. At high pH values, the dissociation of hydroxyl groups (-OH) in oils is suppressed, resulting in decreased solubility. Similar findings have been reported by other studies [3]. Therefore, further experiments were conducted at a fixed pH of 11. 

### 3.2. Effect of Acid Concentration in the Stripping Chamber

**Figure 3:**
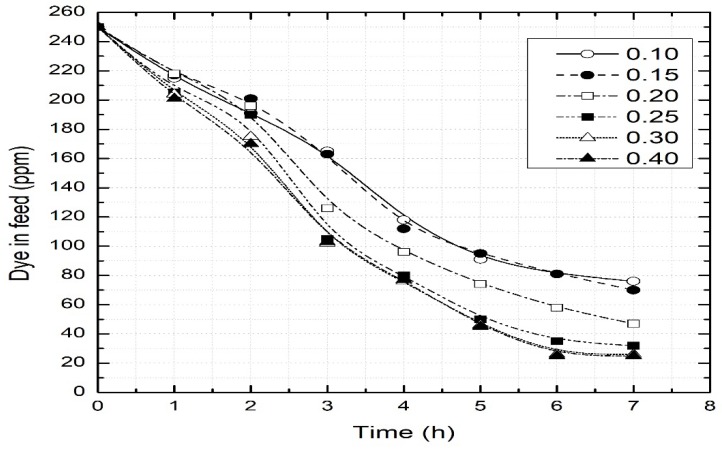
Effect of acid concentration in the stripping chamber. Feed = 100 mg/L dye at pH 11; SLM = sunflower oil; strip = 0.1 M–0.4 M HCl; permeation time = 8 h.

The strength of the solution in the stripping phase plays a significant role in the permeation of the dye. If the dye molecules in the SLM are not stripped off completely, the flux decreases considerably. Therefore, different concentrations of hydrochloric acid in the stripping phase were tried, ranging from 0.1 M to 0.4 M. The results are plotted in Figure 3. It is depicted that increasing acid concentration linearly increases transport until a maximum is achieved. In fact, high acid concentration reduces the dissociation of the oil–dye complex formed within the liquid membrane, thus reaching a maximum value.

### 3.3. Effect of the Dye Concentration

**Figure 4:**
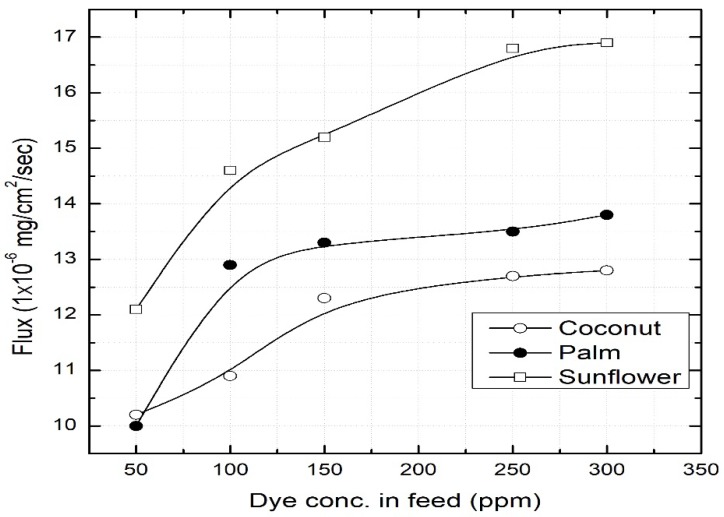
Effect of dye concentration in the feed chamber. Feed = 50–350 mg/L at pH 11; stripping chamber = 0.25 M HCl; permeation time = 8 h.

The change of flux with varying dye concentration in the feed chamber is presented in Figure 4. It is observed that a directly proportional relationship exists between the dye concentration and the flux until a maximum is achieved around 200 mg/L. This is because of the limited surface area of the membrane (14.2 cm^2^), which is responsible for the saturation of the flux. Other studies have reported a similar behavior of the SLM in metalo-organic systems [21]. 

### 3.4. Effect of Mixing Stirrer Speed

**Figure 5:**
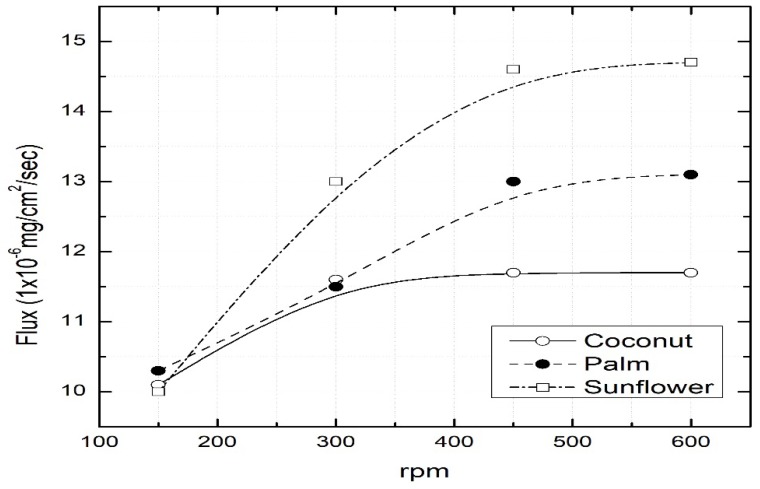
Impact of stirring speed (rpm). Feed = 300 mg/L dye concentration at pH 11; stripping chamber = 0.25 M HCl; permeation time = 8 h.

The speed of the stirrers in the feed and strip chambers plays a significant role in minimizing boundary layer resistance. The effect of stirrer speed on the dye flux is shown in Figure 5. Dye flux improved with increasing stirring speeds until it reached a maximum value of around 550 rpm. Higher speeds caused too much turbulence, which led to the membrane liquid (oil) becoming dislodged from pores. Therefore, the stirring speed was maintained at approximately 500 rpm during all experiments.

### 3.5. Life of Vegetable Oil-Supported Liquid Membrane

**Figure 6:**
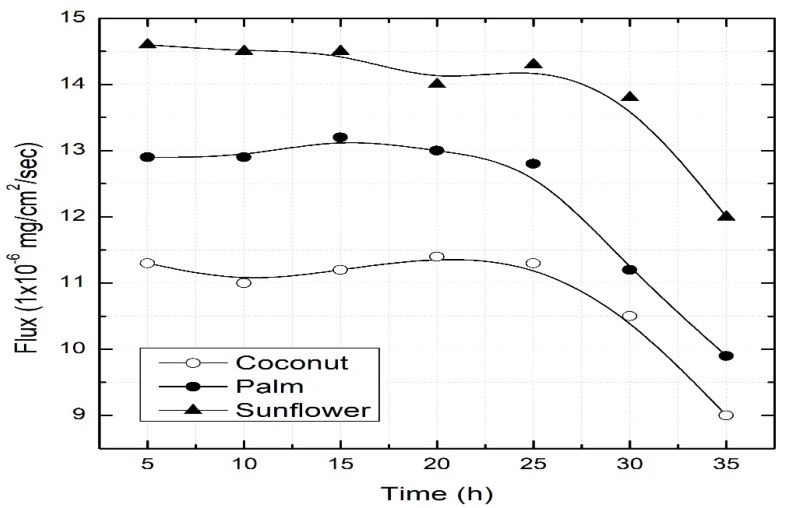
Membrane aging studies. Feed = 150 mg/L dye concentration at pH 11; strip = 0.25 M HCl.

The stability of the PVDF–sunflower oil liquid membrane was studied by conducting long-term permeation experiments. The aging of the SLM is referred to as the loss of membrane liquid from the porous polymeric support. This loss of membrane liquid can be attributed to different factors such as the solubility of the membrane liquid in the feed or stripping solution, pressure gradient or wetting of membrane pores by water [3,4]. An unstable membrane causes a decrease in flux during prolonged use. Therefore, membrane aging experiments were conducted under optimal conditions (Figure 6). It is shown that within 6 h, all dye was transported to the strip side (at 150 mg/L dye concentration). Five experiments were run whereby every 6 h, the feed and strip solutions were refilled but the membrane was not changed. A steady flux was observed for up to 17 h. If there are no morphological changes in the polymeric film, it can be re-impregnated with oil after regular intervals of time. Many factors have been reported that lead to a limited lifespan of liquid membranes, such as Barnard instability, the Margoni effect or membrane materials [22,23].

### 3.6. Permeation Mechanism

**Figure 7:**
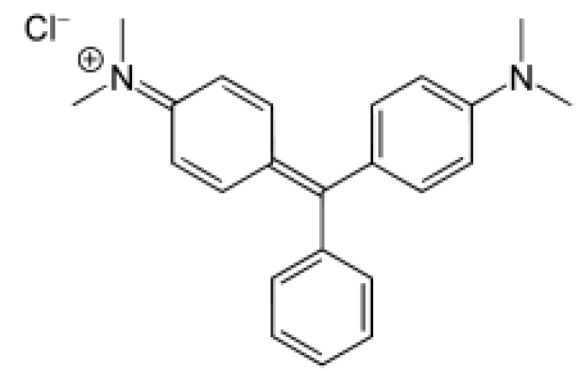
The structural formula of Malachite Green dye.

**Figure 8:**
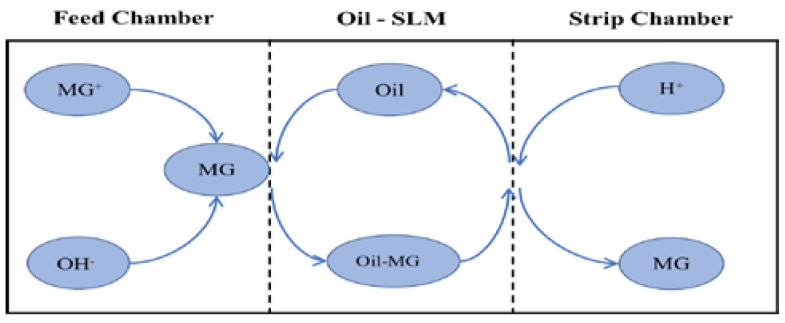
The mechanism of supported liquid membrane transport.

MG is a cationic dye as indicated by its structural formula (Figure 7). The dye cation in aqueous solutions is attracted to the membrane liquid containing the hydroxyl groups (-OH) of vegetable oils. The uphill transport mechanism of MG can be divided into the following steps. At higher pH values in the feed chamber, the dye remains unionized. Unionized dye molecules diffuse into the membrane liquid. In the strip chamber, the unionized dye molecules get ionized owing to the low pH. The dye is stripped in the stripping chamber and the membrane liquid (oil) diffuses back to repeat the cycle (Figure 8).

## 4. Conclusions

Vegetable oils have been successfully used to recover a cationic dye from aqueous solutions. The dye permeation is influenced by numerous variables like the nature of the oil, the pH of the feed and strip chambers, membrane material and stirring speeds. The dye flux increases with increasing dye concentration but reaches a maximum value at higher concentrations. The maximum dye concentration was transported with the following set of parameters: membrane liquid = sunflower oil; pH in feed = 11 ± 0.5; acid concentration in the strip = 0.4 M; initial dye concentration = 200 mg/L; stirring speed = 550 rpm. This lab-scale model can be upgraded to the pilot plant level by increasing the effective surface area of the membrane. This can be done by using hollow fiber modules for the membrane, which increases the throughput many-fold.

## Figures and Tables

**Table 1:** Comparison of dye removal methods from wastewater.

Removal Methods	Advantages	Disadvantages
Photochemical	No sludge formation	By-product formation
Adsorption	Very effective	Expensive on a commercial scale
Electrochemical	Non-toxic end products	High cost of electrical power
Ozonization	Environmentally friendly and no change in wastewater volume	Very short half-life
Membrane Filters	Removes all dyes	Concentrated sludge is generated
Supported Liquid Membranes	Selective removal and cost-effective	Membrane lifetime and aging

**Table 2:** Selected vegetable oils along with their viscosity and flux.

Number	Vegetable Oil	Viscosity (cP)	Flux (mg/cm^2^/sec) × 10^−5^
1	Mustard Oil	96	0.81
2	Virgin Olive Oil	93	0.96
3	Coconut Oil	58	1.53
4	Sunflower Oil	56	1.65
6	Palm Oil	108	0.59
7	Groundnut Oil	117	0.78
8	Castor Oil	485	No permeation
	No oil impregnation		No permeation
